# Marital Status and Place of Death Among Older Adults: A Comparison of European Countries

**DOI:** 10.1093/geroni/igaf122.1317

**Published:** 2025-12-31

**Authors:** Kafayat Mahmoud, Deborah Carr

**Affiliations:** Boston University, Boston, Massachusetts, United States; Boston University, Boston, Massachusetts, United States

## Abstract

Marital status differences in mortality are well-established, but it is unclear how marriage shapes place of death. While most older adults prefer home deaths, hospital or hospice deaths may be necessary for those desiring intensive medical care or palliation, respectively. Associations between marital status and place of death may vary across national contexts, reflecting cultural norms and healthcare policies. We use harmonized data from waves 2 through 9 of SHARE (Survey of Health, Ageing, and Retirement in Europe), to document regional differences in the associations between marital status and place of death (as reported by proxy) in 18 European countries classified into four welfare regimes: Continental (Austria, Belgium, France, Germany, Luxembourg, Netherlands, Switzerland), Eastern (Czech Republic, Estonia, Hungary, Poland, Slovenia), Southern (Greece, Italy, Poland, Spain), and Nordic (Denmark, Sweden). Marital status is not related to place of death in Nordic countries, distinguished by strong social welfare systems. In other regions, we detect strong associations, with slight differences for men and women. For instance, in Continental Europe, never married adults were more likely than married and widowed to die in nursing home whereas widowed adults were more likely to report home deaths relative to married and divorced adults. However, home deaths were especially rare for never married and divorced women. Similarly, in Southern Europe, divorced persons, especially women, were more likely to die in nursing homes or hospice, relative to their married or widowed counterparts, who were more likely to die at home. We discuss the implications for social policy.

